# Asthma Induction During Development and Adult Lung Function, Behavior and Brain Gene Expression

**DOI:** 10.3389/fnbeh.2018.00188

**Published:** 2018-08-30

**Authors:** Jasmine I. Caulfield, Michael J. Caruso, Rebecca A. Bourne, Nicole R. Chirichella, Laura C. Klein, Timothy Craig, Robert H. Bonneau, Avery August, Sonia A. Cavigelli

**Affiliations:** ^1^Department of Biobehavioral Health, Pennsylvania State University, University Park, PA, United States; ^2^The Huck Institutes of the Life Sciences, Pennsylvania State University, University Park, PA, United States; ^3^Center for Brain, Behavior, and Cognition, Pennsylvania State University, University Park, PA, United States; ^4^Allergy, Asthma & Immunology Section, Departments of Medicine and Pediatrics, Penn State University, Hershey, PA, United States; ^5^Departments of Microbiology and Immunology and Pediatrics, Pennsylvania State University College of Medicine, Hershey, PA, United States; ^6^Department of Microbiology and Immunology, Cornell University, Ithaca, NY, United States

**Keywords:** asthma, anxiety, inflammation, house dust mite, methacholine, ultrasonic vocalization

## Abstract

In developing youth, allergic asthma is the most common chronic condition, with 9%–10% of youth affected. Asthma onset during childhood and adolescence is further associated with other health issues, particularly psychiatric conditions. To understand causal mechanisms by which developmental asthma may lead to altered behavior, brain and health trajectories, we developed a mouse model of developmental allergic asthma. In the current study, we tested for potential long-term effects of developmental asthma on adult lung function and behavior and brain gene expression associated with emotion and stress regulation. We manipulated airway inflammation (AI) and methacholine (MCH)-induced bronchospasm (resulting in labored breathing, LB) in young male and female BALB/cJ mice and measured adult outcomes 3 months after final asthma manipulations. Results indicated that allergen exposure, used to cause AI, and which ended on post-natal day 56 (P56), led to persistent lung AI, mucus buildup and gene expression related to allergic asthma 3 months after final allergen exposure. In addition, at this same age, early allergen exposure led to altered brain gene expression related to stress regulation (prefrontal corticotropin releasing hormone receptor 1, *Crhr1* and hippocampal glucocorticoid receptor, *GR*) and serotonin function (brainstem serotonin transporter, *SERT*). On the other hand, LB events during development led to altered anxiety-related behavior. Importantly, sex and pre-asthma fear-related behavior (ultrasonic vocalization, USV rates) modulated these adult outcomes. Asthma that develops during childhood/adolescence may have long-term impacts on emotion and stress regulation mechanisms, and these influences may be moderated by sex and pre-asthma temperament.

Abbreviations: AI, Airway inflammation; AI+LB, Airway inflammation+labored breathing; CON, Control; HDM, House dust mite; IL, Interleukin; LB, Labored breathing; MCH, Methacholine; P, Postnatal day; USV, Ultrasonic vocalization.

## Introduction

Allergic asthma affects 9.5% of children and adolescents in the United States (Akinbami et al., 2012). People with asthma can develop comorbidities with other atopic disorders and other health outcomes (Guerra et al., 2004; Lødrup Carlsen et al., 2014). Importantly, there is a high comorbidity of allergic and internalizing disorders such as anxiety and depression, conditions that are associated with altered stress and immune regulation (Nascimento et al., 2002; Goodwin et al., 2003; Katon et al., 2007; Ross et al., 2007; Buske-Kirschbaum et al., 2008; Tonelli et al., 2009). Interestingly, research suggests that asthma patients are at greater risk for developing these internalizing disorders as early as adolescence (Dudeney et al., 2017).

Serotonin function, in particular transporter function, has been implicated in the pathology of internalizing disorders. For example, serotonin transporter (*SERT*) knockout mice display anxiety-like behavior, and in patients with depression, negative attitudes are correlated with SERT binding potential (Holmes et al., 2003; Meyer, 2007). Interestingly, serotonin also plays a role in allergic responses—its release in the periphery is part of the T-helper type 2 allergic response, and manipulation of peripheral receptors results in improvement of asthma symptoms in murine models (Nau et al., 2015; Shajib and Khan, 2015). In addition, mast cells, which are found in the skin and mucosal tissues as well as in the central nervous system, play an important role in allergic responses and produce molecules like histamine during reactions (Theoharides et al., 2012; Dong et al., 2014). Mast cells are also responsible for producing 20%–40% of the serotonin in the hippocampus, and they produce serotonin in cases of non-allergic asthma and after injury (Nautiyal et al., 2012; Theoharides et al., 2012; Shajib and Khan, 2015). Thus, one mechanism by which allergic asthma may predispose an individual toward internalizing disorders may be by altered serotonin regulation.

Adolescence is an important time for maturation and growth: many changes occur in the body and brain that are critical for normal development of emotion and stress regulation as well as behavior (Spear, 2000; Tirelli et al., 2003; Dahl, 2004; Romeo, 2010, 2015; Sachser et al., 2011; McCormick and Green, 2013). Chronic stressors during this period of growth can have a negative impact on normal development and lead to increased risk of anxiety- or depression-related internalizing disorders (Spear, 2000; Molnar et al., 2001; Barnum et al., 2012; Moretti and Craig, 2013; Dudeney et al., 2017). These adolescent stress effects can also exacerbate adult allergen-induced immune responses and lung hyper-responsiveness, and they can increase midbrain tumor necrosis factor alpha and interleukin (IL)-1 levels following a later immune challenge (Chida et al., 2007; Barnum et al., 2012). The downstream consequences of adolescent stress can be relatively long lasting. For example, chronic adolescent social and non-social stress in male rats and mice can result in anxiety-like effects from 3 weeks to 28 weeks after the end of stress (McCormick et al., 2008; Chaby et al., 2015; Caruso et al., 2017). Chronic adolescent stress can also cause lasting changes in rat hippocampal soma volume 3 weeks after stress completion (Isgor et al., 2004). Thus, adolescence may be a period when organisms are particularly susceptible to long-term effects of stressors, although it is important to note that adolescence may also be a period of stress resilience (Meyer et al., 2016; Sadler and Bailey, 2016).

While evidence suggests that stressors during adolescence predispose an organism toward adult anxiety, it is possible that a predisposition to anxiety prior to adolescence may heighten individual responses to and/or memory of adolescent stressors. In the case of adolescent asthma, an anxious predisposition may exacerbate inflammatory symptoms, resulting in more severe or persistent asthma, and/or more frequent recollection of these symptoms (Richardson et al., 2006). Research indicates that anxiety can be brought on from experiencing a chronic health challenge and associated adverse medical events (Chida et al., 2008). Additionally, parental anxiety can influence a child, putting them at increased risk for developing an anxiety disorder (Whaley et al., 1999). This bi-directional relationship between asthma and internalizing disorders requires further study to elucidate causal directionality and mechanism.

With regard to asthma-internalizing disorder co-morbidity, there are important sex differences to consider. Young males tend to have a higher prevalence of asthma compared to females, but this ratio changes in adolescence and adulthood such that females have higher rates of asthma than males at these older ages (Anderson et al., 1992; Skobeloff et al., 1992; Katon et al., 2007). Other disorders also show distinct sex-specific diagnosis and prevalence patterns. Males tend to show increased rates of behavioral and developmental disorders like attention deficit hyperactivity disorder compared to females, whereas females tend to exhibit higher rates of anxiety, depression and other mood disorders (Andersen and Teicher, 2000; Abikoff et al., 2002; Roza et al., 2003; Holder and Blaustein, 2014). Among adolescents with asthma, females are at greater risk for being diagnosed with anxiety disorders compared to males (Katon et al., 2007; Ross et al., 2007). The effects of adolescent stress can also be worse in females compared to males (Bourke and Neigh, 2011).

In this manuscript, we focus on two features of allergic asthma that may be important in influencing anxiety development. Airway inflammation (AI) is a classic feature of allergic disorders, including asthma, characterized by enhanced T-helper type 2 immune reaction. Allergen-activated T-helper type 2 cells and IL-33 stimulated Type 2 innate lymphoid cells (ILC2) produce cytokines such as IL-4, IL-5 and IL-13 to promote the allergic response and inflammation (Galli et al., 2008; Lloyd, 2010; Salmond et al., 2012; Sjöberg et al., 2017). Certain polymorphisms of IL-33 have also been correlated with increased risk of developing hay fever earlier than 6 years of age (Schröder et al., 2016). Additionally, asthma is characterized by bouts of respiratory dysfunction, bronchoconstriction and labored breathing (LB), which can occur during acute asthma attacks. This state of difficulty breathing that is often associated with decreased oxygen saturation is a significant stressor, and it has been associated with respiratory failure, changes in muscle activity, and remodeling of the airways (Smith and Hudgel, 1980; Ahmad et al., 2012). Both chronic AI and repeated acute LB experiences during development may alter brain and behavior development in such a way as to predispose an individual toward internalizing disorders.

Recent work has established a mouse model of peri-adolescent asthma using independent manipulation of AI and LB to determine how these developmental symptoms affect later-life behavior and physiology. This prior work showed that chronic exposure to intranasal allergen that began during the first week of life led to significant AI, inflammatory cytokine expression, mucus production and collagen buildup in the lungs within 2–4 weeks, and inhaled methacholine (MCH) treatments during development led to significant acute LB events (Saglani et al., 2009; Caulfield et al., 2017). In addition, in adulthood, 3 weeks after termination of repeated peri-adolescent acute LB events, mice exhibited increased anxiety-like behavior and altered brain gene expression (Caulfield et al., 2017). In addition, lung inflammation persisted 3 weeks after cessation of chronic allergen exposure during development, and inflammation and airway hyper-responsiveness was more pronounced in females than males (Blacquière et al., 2010; Caulfield et al., 2017). A longitudinal study on humans with asthma determined that childhood asthma severity (at 7 years of age) strongly predicted lung function and persistence of symptoms in adulthood (at 50 years of age; Tai et al., 2014a). In the current study, we manipulated the same characteristics of allergic asthma (AI and LB) to determine behavioral and physiological effects of these peri-adolescent asthma symptoms 3 months after exposure ended. We used our previously-established mouse model and measured behavior, AI/mucus and brain/lung gene expression 3 months after allergen exposure ended (Caulfield et al., 2017). To determine if long-term effects of developmental asthma were specific to a certain sex or moderated by pre-asthma fearful disposition, we studied both males and females and quantified neonate fear-associated behavior (ultrasonic vocalization, USV) prior to experimental asthma induction.

## Materials and Methods

### Experimental Groups and Design

**Figure 1:**
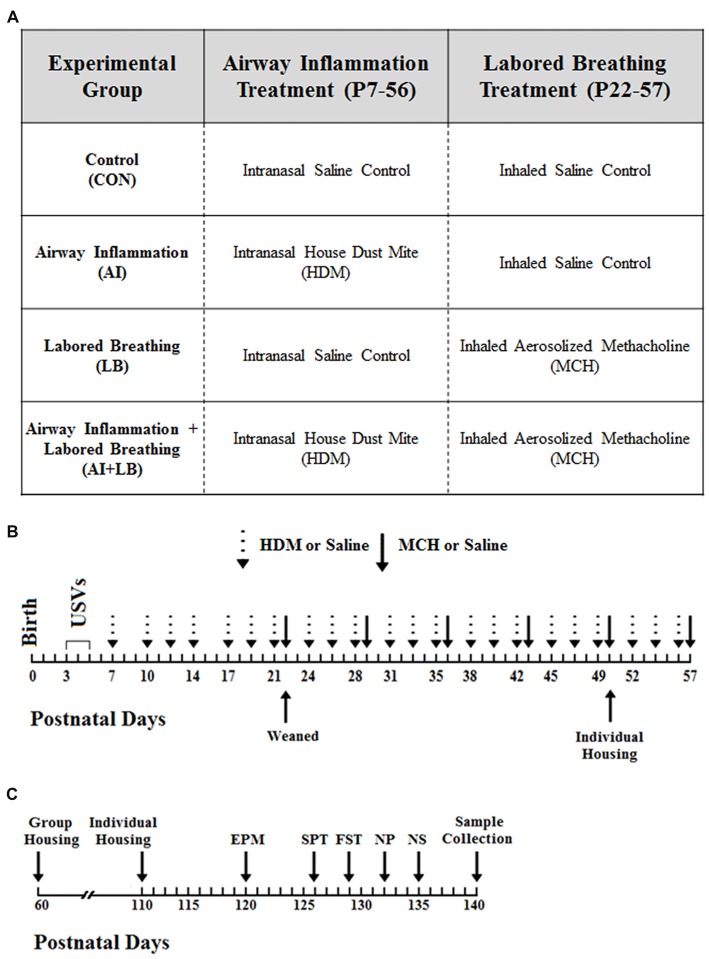
Study Timeline. **(A)** There were four experimental groups in the study, and each group experienced a component of airway inflammation and labored breathing. The first group (Control, CON) served as the control group and received the saline control treatment for both experimental conditions. The second group (Airway Inflammation, AI) was exposed to chronic house dust mite (HDM) to induce inflammation and to the saline control for the labored breathing condition. The third group (Labored Breathing, LB) received saline control for the airway inflammation treatment and methacholine (MCH) to induce labored breathing events. The final group (Airway Inflammation + Labored Breathing, AI+LB) was exposed to both experimental conditions (HDM and MCH). **(B)** Birth was designated as postnatal day (P) zero, and ultrasonic vocalizations (USVs) were conducted from P3–5. AI exposures occurred three times per week from P7–56, and LB treatments occurred once per week from P22–57. **(C)** Mice underwent numerous behavior tests including the elevated plus maze (EPM) on P120, Sucrose Preference Test (SPT) on P126, Forced Swim Test (FST) on P129, Novel Object Task (NP) on P132 and Novel Social (NS) Partner Task on P135. Animals were sacrificed at P140 and samples were collected.

The goal of the present study was to determine what changes persist in mouse adult anxiety-related behavior, gene expression, and corticosterone production 3 months after peri-adolescent asthma symptom exposure. The study used male and female BALB/cJ mice in four peri-adolescent asthma conditions: (1) AI; (2) LB; (3) AI+LB; and (4) Similarly-Handled Controls—CON (Figure 1A). Animals were bred in three cohorts to reach a minimum of 10 animals per sex per condition (*N* = 98, 23–41 mice/cohort). To control for litter effects, same-sex pups from each litter were evenly distributed across all conditions, and all experimental manipulations and data collection were conducted for all littermates at the same time. Body weights were measured on P14, 90 and 140 to determine if the above manipulations altered growth trajectories. By P90, males weighed significantly more than females (P90: *F*_(1,82)_ = 125.7, *p* < 0.001; P140: *F*_(1,81)_ = 411.6, *p* < 0.001), but there were no significant effects of AI, LB, or neonatal USV rates on weight at any age (*F*s < 2.65, *p*s > 0.108). This study was carried out in accordance with the recommendations of the Guide for the Care and Use of Laboratory Animals, Institute for Laboratory Animal Research. The protocol was approved by the Pennsylvania State University Institutional Animal Care and Use Committee.

### Mouse Breeding and Housing

Male and female BALB/cJ breeders were obtained from Jackson Laboratories (Bar Harbor, ME, USA), and mice were bred in the laboratory. To produce litters of sufficient size, sister-pairs were bred with one male to produce “double-litters” (24 double-litters, mean size: 6.5, not culled). Pup identity was tracked by marking them with non-toxic Sharpie^®^ marker until postnatal day (P) 9, at which point all pups were given permanent, unique ear notches. To quantify pre-manipulation fear-related behavior, USVs were measured and coded on P3-P5 (2 min/day) using the “Isolation” method and recording at 65 Hz (Dichter et al., 1996; Brunelli et al., 1997; Branchi et al., 1998; Hahn and Lavooy, 2005; Caulfield et al., 2017). Several inbred strains of mice, including BALB/cJ mice, display peak USV production at postnatal day P3 with USVs in the range of 60–80 kHz, and the amount of calling decreases during the first 2 weeks of life (Bell et al., 1972). In rodents, USVs can predict later-life emotion regulation; mice selectively bred based on pup frequency of USVs develop into distinct high and low-calling lines, and offspring of low-calling lines demonstrate less anxiety-like behavior compared to the high-calling line (Dichter et al., 1996; Brunelli et al., 1997). In the present study, pups within each litter were characterized as either high or low calling relative to their litter median, and high- and low-calling pups were evenly distributed among treatment conditions.

Pups were weaned from the dam at P22 and placed into same-sex sibling groups (2–4 mice) in standard cages (28 cm × 17 cm × 12 cm) with corn-cob bedding. Cages were not individually ventilated, but rather had standard wire lid covers with a filter top over the lid. Identical weekly husbandry procedures were used for all groups. Mice remained in these groups until P50, at which point they were single-housed in standard cages, then returned to cages with their original littermates on P60. On P110, mice were again single-housed for behavior testing (P120–135) until sacrificed (P140; Figure 1C). All mouse cages had a red polypropylene tube, which acted as environmental enrichment and a familiar transport vehicle for experimental manipulations (Roy et al., 2001). Throughout the study, colony rooms were maintained at 21 ± 1°C, at 30%–70% humidity, and on a reverse 12:12 light:dark schedule (lights on 18:00 h, lights off 06:00 h). All animals had *ad libitum* access to food and water throughout the study.

### Induction of Adolescent Allergic Asthma Symptoms

Experimental procedures for induction of allergic asthma symptoms were conducted as previously detailed and are briefly described below (Caulfield et al., 2017).

#### Airway Inflammation (AI)

AI was induced by regularly exposing young mice intranasally to an extract of the most common aeroallergen for humans—house dust mite (*Dermatophagoides pteronyssinus*, HDM; Greer Labs, NC, USA). The AI and AI+LB groups were exposed intranasally to a solution of HDM three times per week, and the CON and LB groups received saline on the same schedule using the same technique (Figure 1B). From P7–15, mice received 10 μg (10 μL of 1 mg/ml protein weight solution in saline) of HDM at each exposure, and from P16–56, doses increased to 15 μg HDM (15 μL) and were administered under brief isoflurane anesthesia. This method leads to significant lung inflammation within 2 weeks of first dosage, and elevated inflammation persists throughout the exposure period and at least 3 weeks after cessation of the HDM exposures (Saglani et al., 2009; Caulfield et al., 2017).

#### Labored Breathing (LB)

LB was induced by exposure to inhaled methacholine (MCH; Sigma, St. Louis, MO, USA), a muscarinic receptor agonist. From P22–57 exposures occurred once per week (Figure 1B). Mice were placed in a whole-body plethysmograph holding chamber (7.5 cm diameter × 7 cm height; Data Sciences International, New Brighton, MN, USA) and allowed to acclimate for 3 min followed by baseline breathing recorded for 3 min. After acclimation and baseline, LB mice were exposed to five increasing doses of aerosolized MCH (0, 6.25, 12.5, 25 and 50 ng/ml in 100 μl saline). AI+LB mice received a half-dose of MCH (0, 3.13, 6.25, 12.5, 25 ng/ml in 100 μl saline) to arrive at LB estimates comparable to LB mice. In prior work, we titrated the best MCH doses for the LB and AI+LB groups to arrive at similar level of LB in both groups. CON and AI mice experienced the exact same procedures but received saline instead of MCH. To verify and estimate extent of bronchoconstriction, enhanced pause (Penh) was recorded (Hamelmann et al., 1997) using FinePointe software. Behavior in the plethysmograph was recorded throughout each session (active, sit still, hunch, LB, drool, gape). If three Penh values were above 15 or if a mouse was visibly distressed, the MCH administration procedure was terminated early. We have previously demonstrated that this procedure leads to significant LB events in both allergen-exposed and -unexposed BALB/cJ mice (Caulfield et al., 2017).

### Behavior Testing

#### Anxiety-Related Behavior, Elevated Plus Maze (EPM)

On P120, elevated plus maze (EPM) was conducted to measure anxiety behavior. This test has been pharmacologically validated, and it is a classic test for observing anxiety-related behavior in mice (Pellow et al., 1985; Lister, 1987; Hogg, 1996; File, 2001; Carobrez and Bertoglio, 2005). The maze consists of two open (30 × 5 cm) and two closed (30 × 14.5 × 5) flat perpendicular arms elevated 42 cm above the ground. Test orders were pseudo-randomized to balance conditions and litter mates. Mice were brought to the test room ~1 h before testing, transported to the maze in the familiar red tube, and placed in the maze facing an open arm. Testing was completed under red light illumination (<5 lux), and behavior video-recorded for 5 min. Entry into a maze arm was defined as four limbs crossing the boundary between sections. Videos were scored for: percent time spent on open arms, total number of entries into open arms, and total number of entries into open and closed arms. Percent time on and entries into the open arms were used as inverse metrics of anxiety-like behavior, and total arm entries were used to measure overall locomotion.

#### Hedonic Behavior, Sucrose Preference Test (SPT)

On P126, free-choice consumption of sucrose was recorded in the Sucrose Preference Test (SPT) to examine mouse hedonic behavior (Strekalova et al., 2004). SPT is a reliable measure of depression-related hedonic behavior (Porsolt et al., 1977). Mice had 24-h free access to a bottle with tap water and another bottle with a 3% sucrose solution. BALB/cJ mice show a preference for sucrose solutions with a concentration of sugar that is higher than other inbred mouse strains (Lewis et al., 2005). After 12 h, bottle positions were switched to avoid side preference biases. Prior to and following the 24-h period, each bottle was weighed to calculate consumption of sucrose solution relative to water. Decreased relative sucrose consumption was used as an index of anhedonic behavior (McCormick and Green, 2013).

#### Depression-Related Behavior, Forced Swim Test (FST)

Forced Swim Test (FST) was conducted on P129 to measure depression-related behavior. Mice were individually tested by placing them into a large beaker of water (25–27°C) for 6 min. Latency to become immobile, number of times immobile, and total time immobile were quantified from video coding. Immobility was defined as lack of movement in at least three limbs. FST is a classic test for depressive behaviors, and higher levels of immobility are indicators of this (McCormick and Green, 2013).

#### Novelty Exploration, Novel Physical and Novel Social Arenas

Exploratory behavior was measured on two separate arenas, one containing novel mouse-sized objects and another containing a novel social (NS) partner as previously described (Cavigelli et al., 2007). Briefly, both arenas were 120 cm × 120 cm with opaque walls and a Plexiglas cover. The floor was covered with semi-soiled bedding. For the Novel Physical test, small objects were placed in three of the four corners. For the NS test, a same-age, same-sex mouse was placed in a wire container in one corner, and a similar empty container was placed in the opposite corner. For both tests, mice were run individually by carrying them in a red enrichment tube from their home cage to the open corner of the arena. Behavior was video-recorded using a camera positioned well above the arena, and all testing was conducted in low, red light (<10 lux). Latency to approach a novel object or the NS partner were recorded in each arena; these behaviors are associated with stress regulation (Cavigelli et al., 2007).

### Physiological Outcomes

#### Lung Inflammation, Mucus and Collagen

Left and right posterior lung sections were collected, preserved in formalin, embedded in paraffin and then sliced. Consecutive slices were stained with periodic acid-Schiff, hematoxylin and eosin (H&E), or Masson’s trichrome to quantify mucus, inflammation and collagen respectively. Mucus levels were quantified on a scale of 0–6 as previously described, with increasing numbers indicating increasing mucus (Caulfield et al., 2017), and an average mucus score was calculated per mouse based on measures from six slices. The number of discrete inflammation areas (clusters of inflammatory cells) and the length of each area were measured perpendicular to airway/vessel membranes (20 μm diameter or larger) as previously described (Caulfield et al., 2017). Total number of these areas and mean length were calculated from the six largest areas (three from each lung/mouse, or as many areas as possible). Average collagen thickness was quantified for each mouse based on five thickness measures from each of 3–5 airways on each of two lung slices per mouse as previously detailed(Caulfield et al., 2017).

#### Adult Lung Cytokine Gene Expression

Lungs were collected at sacrifice and stored in RNAlater (Ambion, Carlsbad, CA, USA) for 24-h before freezing at −80°C. Tissue RNA extraction was conducted using TRIzol reagent (Invitrogen; Carlsbad, CA, USA) and Qiagen RNeasy columns (Qiagen, Germantown, MD, USA). RNA quantity and quality were determined with a NanoDrop™ spectrophotometer (Thermo Fisher Scientific, Wilmington, DE, USA) and Agilent 2100 BioAnalyzer™ (Agilent Technologies, Santa Clara, CA, USA), respectively. Complementary DNA (cDNA) was reverse transcribed from RNA with High-Capacity cDNA Reverse Transcription kits (Applied Biosystems, Wilmington, DE, USA). Quantitative real time PCR (qRT-PCR) was conducted to measure relative abundance of the following genes in cDNA: *IL-4* (Mm00445259 m1), *IL-5* (Mm00439646 m1). We also conducted PCR to measure *IL-13* expression (Mm00434204 m1), but because of poor amplification, we do not report these results here. Reactions were prepared in 96-well plates in triplicate with validated TaqMan probes on a StepOnePlus RT PCR System (Applied Biosystems). The following cycle settings were implemented: 50°C for 2 min, 95°C for 10 min, 40 cycles of 95°C for 15 s and 60°C test for 60 s. Beta actin (*Actb*) was used as the reference gene. Gene expression scores were standardized to the median control mouse, and relative gene abundance in each sample was determined with the 2^−ΔΔCT^ method as has been done previously (Caulfield et al., 2017).

#### Adult Brain Serotonin- and HPA-Related Gene Expression

Brains were freshly dissected at sacrifice, and the following brain regions were collected: brainstem, hippocampus and prefrontal cortex (PFC). All sections were collected, processed and analyzed as described above for lung cytokine gene expression and as described previously (Caulfield et al., 2017). The following TaqMan Gene Expression Assay primers and probes were used for PCR with brain tissue cDNA: *SERT*, serotonin receptor 1a (*5Htr1a*), corticotropin releasing hormone receptor 1 (*Crhr1*) and glucocorticoid receptor (*GR*; Life Technologies, Mm00439391 m1, Mm00434106 s1, Mm00432670 m1 and Mm00433832 m1, respectively). The serotonin system is highly implicated in anxiety and depression-related disorders (Holmes et al., 2003), and this system is known to be affected in models of allergy (Nau et al., 2015; Shajib and Khan, 2015). Corticotropin releasing hormone and GRs are important aspects of the stress response and anxiety-related behavior, and their function can also become altered in response to developmental stress (Contarino et al., 1999; Spear, 2000; McCormick and Green, 2013).

#### Serum Corticosterone

To measure basal glucocorticoid levels, trunk blood was collected immediately after sacrifice. Mean time required to sacrifice and collect a blood sample after removal from the home cage was 4.3 min (SEM: 0.11). Samples were centrifuged at 15,000 rpm for 15 min at 4°C, and serum collected and stored at −80°C until analysis. Samples were analyzed in duplicate with a commercial [125I] radioimmunoassay kit (MP Biomedicals, Solon OH, USA). Intra-and inter-assay coefficients of variation for a low and high control were 4.72 and 6.92 (for low control) and 5.51 and 6.88 (for high control). Time required for sample collection was not related to serum corticosterone concentration (*r* = −0.125, *p* = 0.182).

### Statistical Analyses

To compare behavioral and physiological outcome variables across conditions, ANCOVAs were conducted with AI (intranasal saline vs. HDM exposure), LB (inhaled aerosolized saline vs. MCH exposure), Sex (male vs. female) and USV category (high vs. low) as factors. We used the cohort mean for each outcome variable as a covariate to control for variation between cohorts. Alpha was designated as 0.05. For all statistical tests, variable distribution was examined to verify normal distribution. The following variables were log transformed to achieve a normal distribution for analyses: lung *IL-4* and *IL-5* gene expression, brainstem *SERT* gene expression, serum corticosterone, percent sucrose consumed in the SPT, percent time spent on the open arms of the EPM, latency to immobility in the FST, percent time immobile in the FST, latency to approach an object in the Novel Physical task, and latency to approach a social partner in the NS task. Outliers were defined as ±2.5 SD and removed prior to statistical analyses. Figures detail the untransformed estimated marginal means for clarity. When there were no main or interaction effects of Sex or USV category, we presented means in the figures collapsed across these factors. Repeated measures ANOVAs were used to determine if LB and/or Penh values changed during repeat administrations from P22–57. Correlation analyses were conducted to determine if there were any linear relationships between gene expression (lung or brain), lung function measures and behavioral outcomes.

## Results

### Adult Physiology (P140)

#### Lung Cytokine Gene Expression

**Figure 2:**
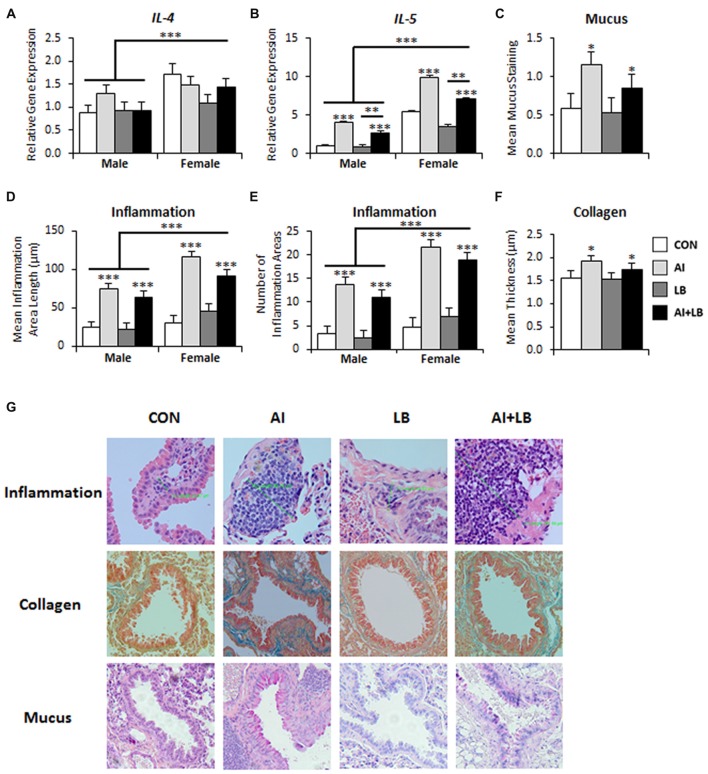
Long-term lung effects of peri-adolescent allergen exposure. **(A)** Interleukin (*IL*)*-4* gene expression was significantly elevated in females compared to males. **(B)** *IL-5* gene expression demonstrated main effects of sex, AI and LB. **(C)** Mucus levels, quantified from PAS-stained lung sections, were elevated in animals that received AI. **(D,E)** Lung inflammation, quantified from hematoxylin and eosin (H&E)-stained sections, indicated that the average inflammation area length **(D)** and number of areas **(E)** were significantly elevated in females compared to males and in AI animals compared to non-AI animals. **(F)** Collagen levels, quantified from Mason trichrome-stained sections, were elevated in animals that received AI. **(G)** Representative images of lung sections stained for inflammation (H&E), mucus (PAS) and collagen (Mason trichrome). Magnification is 20×. *p < 0.05, **p < 0.01, ***p < 0.001.

Three months after the final adolescent HDM and MCH exposures, there was a significant main effect of Sex on *IL-4* and *IL-5* expression—females had elevated levels compared to males (*IL-4*:* F*_(1,69)_ = 11.13, *p* < 0.001, Figure 2A; *IL-5*: *F*_(1,68)_ = 158.23, *p* < 0.001, Figure 2B). There were no other significant main effects or interactions present for *IL-4* (*F*s < 3.62, *p*s > 0.061). There was a main effect of AI on *IL-5* expression—animals treated with HDM had higher *IL-5* expression than those not treated with HDM (*F*_(1,69)_ = 90.24, *p* < 0.001, Figure 2B). There was also a main effect of LB, where MCH-treated mice had less *IL-5* expression compared to mice that were not exposed to MCH (*F*_(1,68)_ = 9.30, *p* < 0.01, Figure 2B). Finally, there was a significant main effect of USV, where high-calling mice had lower *IL-5* expression than low-calling mice (*F*_(1,68)_ = 4.41, *p* < 0.05; data not shown in figure). No significant interactions were noted for *IL-5* (*F*s < 2.02, *p*s > 0.160). We had poor amplification for *IL-13* and thus do not report results here.

#### Lung Mucus

There was a significant effect of AI on mucus levels; mice exposed to chronic peri-adolescent HDM treatments had higher levels of mucus in the lungs 3 months after final allergen treatments (*F*_(1,78)_ = 6.07, *p* < 0.05, Figure 2C,G). No other main effects or interactions were significant (*F*s < 3.63, *p*s > 0.060).

#### Lung Inflammation

Three months following completion of allergen exposure, mice treated with HDM still had significant symptoms of AI—i.e., greater average length and number of discrete areas of inflammation—compared to non-HDM treated mice (AI main effect on inflammation area length—*F*_(1,78)_ = 82.76, *p* < 0.001; and inflammation area number—*F*_(1,78)_ = 100.70, *p* < 0.001, Figures 2D,E,G). There was also a main effect of Sex and an interaction of Sex and AI for measures of AI (Sex effect: average length of inflammation area—*F*_(1,78)_ = 16.03, *p* < 0.001, average number of inflammation areas—*F*_(1,78)_ = 20.93, *p* < 0.001, Figures 2D,E; Sex × AI interaction: average number of inflammation areas—*F*_(1,78)_ = 4.43, *p* < 0.05). Females had greater inflammation than males, and female-specific increased inflammation was particularly pronounced in the HDM-treated mice. There were no other significant main or interaction effects for average inflammation area length or count (*F*s < 3.86, *p*s > 0.053; *F*s < 2.04, *p*s > 0.157).

#### Lung Collagen

Mice that were exposed to developmental HDM had significantly more collagen compared to mice that were not exposed to HDM (*F*_(1,71)_ = 4.12, *p* < 0.05; Figures 2F,G). There were no other significant main or interaction effects for average collagen thickness (*F*s < 3.68, *p*s > 0.059).

### Peri-adolescent Bronchoconstriction (P22–57)

**Figure 3:**
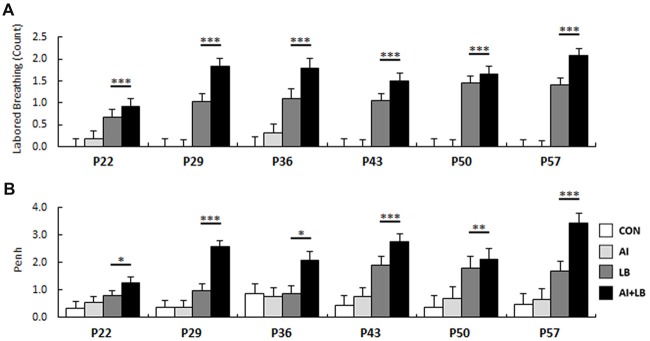
Developmental bronchoconstriction. Mean **(A)** LB response and **(B)** Penh value for mice exposed to MCH was significantly increased compared to mice that were exposed to saline. This was evident at each age of administration. *p < 0.05, **p < 0.01, ***p < 0.001.

Compared to saline administration, MCH administration caused significantly increased LB counts and Penh values throughout development (LB: *F*_(1,20)_ = 170.56, *p* < 0.001, Figure 3A; Penh: *F*_(1,20)_ = 130.28, *p* < 0.001, Figure 3B). LB and Penh values increased with age (LB: *F*_(5,350)_ = 3.66, *p* < 0.01; Penh: *F*_(1,100)_ = 3.15, *p* < 0.05), and mice treated with both HDM and MCH (AI+LB) had greater increases in LB and Penh values than mice treated with MCH alone (LB; AI × LB interaction: LB—*F*_(1,20)_ = 4.47, *p* < 0.05; Penh—*F*_(1,20)_ = 15.32, *p* < 0.001; Group means across all ages for LB: CON 0.00 ± 0.19, AI 0.08 ± 0.16, LB 1.12 ± 0.18, AI+LB 1.63 ± 0.18; Penh: CON 0.48 ± 0.32, AI 0.63 ± 0.32, LB 1.34 ± 0.30, AI+LB 2.37 ± 0.30).

### Adult Behavior

#### Elevated Plus Maze (P120)

**Figure 4:**
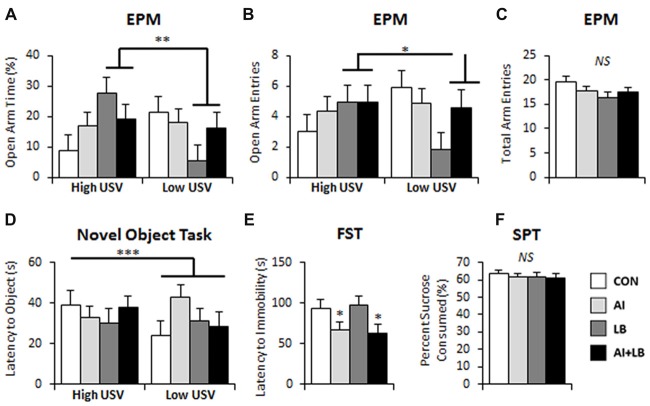
Long-term behavioral effects of peri-adolescent allergic asthma symptoms. **(A,B)** High-calling mice (based on USV) that also experienced developmental LB showed less anxiety-like behavior on the EPM compared to low-calling mice in terms of time spent on the open arms of the maze **(A)** and number of entries made into the open arms of the maze **(B)**. **(C)** There were no significant differences evident in number of total arm entries made in the EPM. **(D)** In the novel object task, low-calling mice demonstrated a faster latency to approach a novel object compared to mice that were categorized as high-callers. **(E)** AI animals demonstrated faster latency to immobility in the FST compared to those that did not experience AI. **(F)** No significant main effects were observed in the sucrose preference test. *p < 0.05, **p < 0.01, ***p < 0.001, *NS*, not significant.

There was a significant interaction of LB and USV on percent time and number of entries in the open arms of the EPM. For high-calling mice, MCH-exposure led to more time spent and more entries in the open arms. For low-calling mice, MCH-exposure led to less time spent and fewer entries into the open arms relative to unexposed mice (LB × USV interaction: percent time in open arms—*F*_(1,80)_ = 8.05, *p* < 0.01; and number of entries to open arms—*F*_(1,80)_ = 4.97, *p* < 0.05, Figures 4A,B). Additionally, there was a three-way interaction between AI, LB and USV for time spent on the open arms, where the MCH effect described above was dampened in mice exposed to both HDM and MCH (*F*_(1,80)_ = 5.01, *p* < 0.05, note final AI+LB bar in Figure 4A). No other significant main or interaction effects were observed for time spent or entries on open arms of EPM (*F*s < 2.79, *p*s > 0.099;* F*s < 2.81, *p*s > 0.098). Time spent on the open arms and number of entries on open arms were significantly correlated (*r* = 0.782, *p* < 0.001). No significant main effects or interactions were observed in total arm entries in the EPM (*F*s < 2.98, *p*s > 0.088; Figure 4C).

#### Latency to Approach Novelty (P132, P135)

In the novel object test (P132), there was a significant main effect of Sex—males took longer to approach a novel object compared to females (*F*_(1,66)_ = 4.97, *p* < 0.05; Figure 4D). There was also a significant three-way interaction between AI, LB and USV (*F*_(1,81)_ = 4.15, *p* < 0.05); HDM and MCH independently decreased adult approach latencies for high-calling mice and increased latency time in low-calling mice. Both effects were negated by exposure to both HDM and MCH. There were no other significant effects or interactions between groups to approach a novel object (*F*s < 1.32, *p*s > 0.255). There were no significant main effects or interactions in latency to approach a novel partner (P135; *F*s < 2.29, *p*s > 0.135).

#### Forced Swim Test (P129)

Adult mice that were exposed to HDM during development became immobile faster in the FST compared to mice that were not exposed (*F*_(1,80)_ = 5.68, *p* < 0.05, Figure 4E). There were no other main or interaction effects for latency to immobility (*F*s < 2.51, *p*s > 0.117). There were no significant main effects or interactions for percent time spent immobile in the FST (*F*s < 2.64, *p*s > 0.108). There was a significant three-way interaction between Sex, AI and USV for number of times immobile (*F*_(1,81)_ = 5.37, *p* < 0.05). HDM-exposure caused high-calling males and low-calling females to increase the number of immobility bouts in the FST compared to similar calling males and females that were not exposed to HDM. No other main effects or interactions were found for number of times immobile (*F*s < 2.77, *p*s > 0.100).

#### Sucrose Preference Test (P126)

Analysis of percent sucrose consumed in the SPT revealed a significant interaction between Sex and USV; high-calling female mice consumed less sucrose than low-calling females, whereas high-calling males consumed more sucrose solution than low-calling males (*F*_(1,80)_ = 4.09, *p* < 0.05). No other main effects or interactions were observed for percent sucrose consumed (*F*s < 2.54, *p*s > 0.115; Figure 4F).

### Basal Corticoid Rhythm (P140)

Circulating basal corticosterone levels were measured at time of sacrifice (P140), 3 months following HDM/MCH exposure. There were no main or interaction effects on adult circulating corticosterone concentrations (*F*s < 2.44, *p*s > 0.123; experimental group means: CON 47.53 ± 12.48, AI 48.88 ± 11.14, LB 40.01 ± 12.32, AI+LB 55.82 ± 12.08).

### Adult Brain Gene Expression (P140)

**Figure 5:**
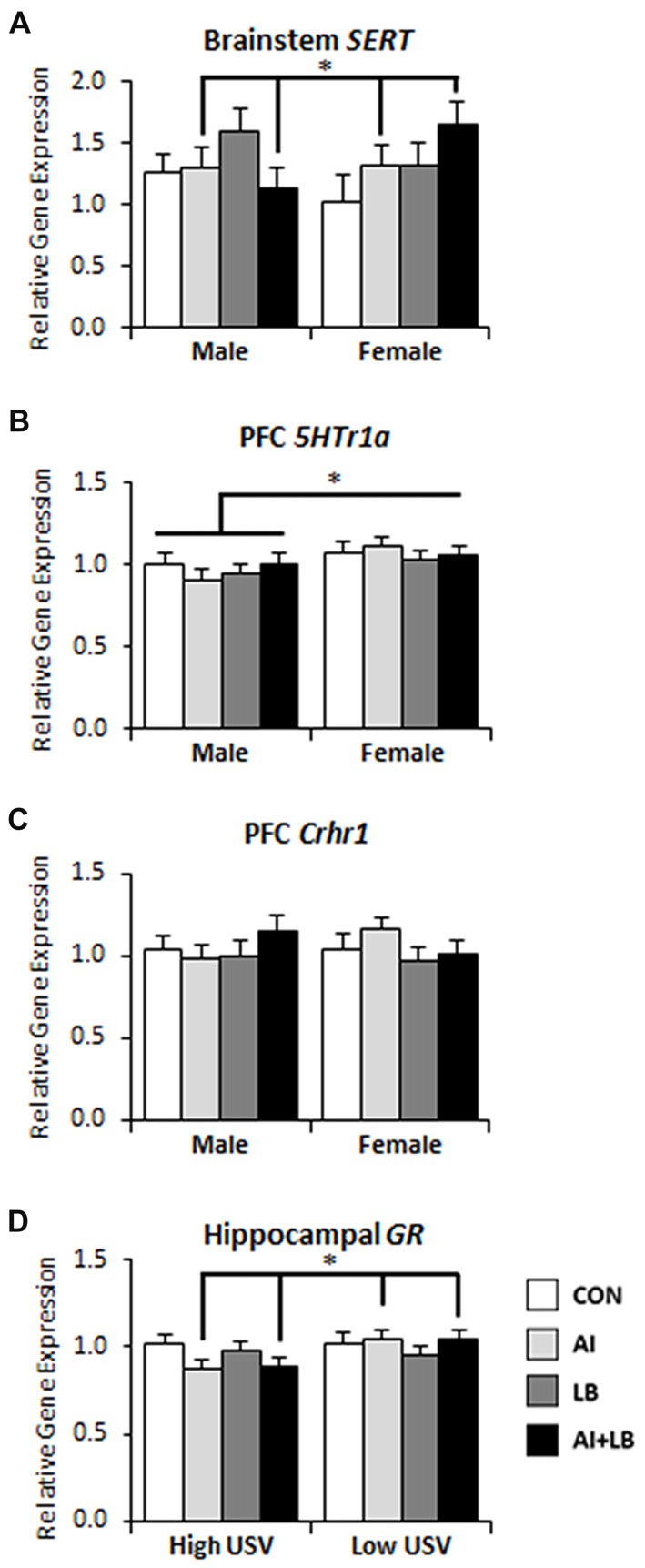
Long-term brain gene expression effects of peri-adolescent allergic asthma symptoms. **(A)** Females that experienced developmental AI had higher serotonin transporter (*SERT*) gene expression in brainstem compared to males that experienced AI in development. **(B)** Females had higher levels of serotoninreceptor 1a (*5Htr1a*) gene expression in prefrontal cortex (PFC) compared to males 3 months after asthma symptom exposures had been completed. **(C)** PFC corticotropin releasing hormone receptor 1 (*Crhr1*) gene expression in male and female mice across asthma condition; HDM-treated low-calling females had more expression than HDM-treated low-calling males (this three-way interaction is not indicated in the figure). **(D)** High-calling mice that experienced AI had decreased glucocorticoid receptor (*GR*) gene expression in hippocampus compared to low-calling mice that experienced AI. *p < 0.05.

Three months after the end of peri-adolescent asthma treatments, females that had been exposed to HDM during development had greater *SERT* expression in the brainstem, whereas HDM-exposed males had diminished *SERT* expression compared to non-HDM exposed mice (Sex × AI interaction—*F*_(1,81)_ = 6.53, *p* < 0.05, Figure 5A). There was also a three-way interaction of AI, LB and USV, such that high-calling pups exposed to either HDM or MCH showed increased *SERT* expression, whereas low-calling pups had increased *SERT* expression only if they had received both HDM and MCH (*F*_(1,81)_ = 4.70, *p* < 0.05). There were no other main or interaction effects on *SERT* expression (*F*s < 2.72, *p* > 0.103). Females had higher *5HTr1a* expression in PFC than males (*F*_(1,69)_ = 5.31, *p* < 0.05, Figure 5B). No other main effects or interactions were significant for PFC *5HTr1a* expression (*F*s < 2.10, *p* > 0.151). For hippocampal *5HTr1*a expression, there were no main or interaction effects (*F*s < 1.38, *p* > 0.244). There was a significant three-way interaction of Sex, AI and USV on *Crhr1* expression in PFC; relative to control mice, HDM-treated low-calling females had greater expression than HDM-treated low-calling males (*F*_(1,67)_ = 5.02, *p* < 0.05, Figure 5C; three-way interaction not shown on figure). No other interactions or main effects were significant (*F*s < 2.11, *p* > 0.151). For high-calling mice, developmental HDM exposure resulted in decreased hippocampal *GR* expression in adulthood, whereas the reverse was true for low-calling mice (AI × USV interaction—*F*_(1,69)_ = 5.52, *p* < 0.05, Figure 5D). No other main effects or interactions were significant (*F*s < 3.90, *p* > 0.052).

### Correlations

#### Lung Measures

Many of the measures of lung inflammation and function were significantly correlated with one another (correlation statistics in Table 1). *IL-5* expression was significantly and positively correlated with average inflammation area length (*r* = 0.694, *p* < 0.001), inflammation area count (*r* = 0.661, *p* < 0.001) and mucus (*r* = 0.275, *p* = 0.011). Average inflammation area length was strongly and positively correlated with inflammation area count (*r* = 0.913, *p* < 0.001), and mucus was positively correlated with inflammation area length and count (*r* = 0.395, *p* < 0.001; *r* = 0.359, *p* < 0.001). Collagen was positively correlated with inflammation patch count (*r* = 0.211, *p* = 0.050), but it was not correlated with average patch length (*r* = 0.188, *p* = 0.140), mucus (*r* = 0.188, *p* = 0.082), or *IL-5* gene expression in lungs (*r* = 0.112, *p* = 0.333).

**Table 1:** Statistical correlations among lung physiology, gene expression and behavior.

Factor 1	Factor 2	Sample size	Pearson correlation (*r*)	Significance (*p*)
Lung *IL-5* Expression	Inflammation Area Length	85	0.694	**<0.001**
Lung *IL-5* Expression	Inflammation Area Count	85	0.661	**<0.001**
Lung *IL-5* Expression	Mucus	85	0.275	**0.011**
Inflammation Area Count	Inflammation Area Length	95	0.913	**<0.001**
Mucus	Inflammation Area Length	95	0.395	**<0.001**
Mucus	Inflammation Area Count	95	0.359	**<0.001**
Collagen	Inflammation Area Count	87	0.211	**0.050**
Collagen	Inflammation Area Length	87	0.160	0.140
Collagen	Mucus	87	0.188	0.082
Collagen	*IL*-5 Gene Expression	77	0.112	0.333
EPM % Time on Open Arms	Inflammation Area Length	94	0.213	**0.039**
EPM % Time on Open Arms	Inflammation Area Length	94	0.187	0.071
EPM % Time on Open Arms	Mucus	94	0.324	**0.001**
EPM Open Arm Entries	Inflammation Area Length	94	0.212	**0.040**
EPM Open Arm Entries	Inflammation Area Length	94	0.220	**0.033**
EPM Open Arm Entries	Mucus	94	0.232	**0.025**
FST Latency to Immobility	Inflammation Area Length	95	−0.046	0.661
FST Latency to Immobility	Inflammation Area Count	95	−0.086	0.408
FST Latency to Immobility	Mucus	95	−0.001	0.993
FST Latency to Immobility	*IL*-5 Gene Expression	85	0.155	0.157
PFC *5HTr1a* Expression	EPM % Time on Open Arms	85	0.324	**0.002**
PFC *5HTr1a* Expression	EPM Open Arm Entries	85	0.234	**0.031**
PFC *5HTr1a* Expression	Mean USV Calling Rate	102	−0.294	**0.003**
Hippocampus *5HTr1a* Expression	EPM % Time on Open Arms	85	0.108	0.326
Hippocampus *5HTr1a* Expression	EPM Open Arm Entries	85	0.095	0.386
Hippocampus *5HTr1a* Expression	Mean USV Calling Rate	103	0.163	0.100

*Bold values indicate significant correlations*.

#### Lungs and Anxiety- and Depression-Related Behavior

Some lung measures were significantly correlated with behavior (Table 1). Percent time spent on the open arms of the EPM was positively correlated with average inflammation area length (*r* = 0.213, *p* < 0.05) and mucus (*r* = 0.324, *p* < 0.001), and marginally related to inflammation area count (*r* = 0.187, *p* = 0.071). Open arm entries on the EPM were positively correlated with average inflammation area length (*r* = 0.212, *p* < 0.05), inflammation area count (*r* = 0.220, *p* < 0.05), and mucus (*r* = 0.232, *p* < 0.05). However, there were no significant correlations between depression-related behavior (latency to immobility on the FST) and the following lung measures: average inflammation area length (*r* = −0.046, *p* = 0.661), number of inflammation areas (*r* = −0.086, *p* = 0.408), mucus (*r* = −0.001, *p* = 0.993) and lung *IL-5* expression (*r* = 0.155, *p* = 0.157).

#### Brain Gene Expression and Anxiety-Related Behavior

A few correlations were found between brain gene expression and anxiety-related behavior (Table 1). *5HTr1a* gene expression in PFC was significantly positively correlated with percent time spent on the open arms of the EPM (*r* = 0.324, *p* < 0.01) and number of open arm entries in the EPM (*r* = 0.234, *p* < 0.05). It was also negatively correlated with mean USV calling rate (*r* = −0.294, *p* < 0.01). On the other hand, *5HTr1a* expression in the hippocampus was not correlated with these same behavioral measures (*r* = 0.108, *p* = 0.326; *r* = 0.095, *p* = 0.386; *r* = 0.163, *p* = 0.100).

## Discussion

### Long-Term Behavior and Brain Changes Following Peri-adolescent Asthma

Results of the current study indicate that chronic inhaled allergen exposure during development led to long-term changes in lung function. Exposure to HDM extract three times per week from neonatal age to late adolescence led to increased AI, mucus, collagen and *IL-5* gene expression 3 months after final allergen exposure, particularly in females. In addition, developmental allergen exposure (and associated lung alterations) altered gene expression for brainstem *SERT* and PFC *Crhr1*, with these effects being sex- and USV-specific. Females that had been exposed to allergen during development showed increased brainstem *SERT* expression, and low-calling females showed increased PFC *Crhr1* expression, compared to non-exposed females. Allergen-exposed males, on the other hand, showed decreased brainstem *SERT* expression and more modest increases in PFC *Crhr1* expression compared to unexposed males. These results, 3 months after final asthma symptom induction, contrast with previously observed results on short-term responses to developmental allergen exposure. Specifically, in a prior study, we found that 3 weeks after symptom induction was completed, there were no significant effects of HDM exposure on similar adult anxiety- or depression-related behaviors or similar brain gene expression related to emotion and stress regulation. Rather, weekly exposure to MCH to induce LB led to adult anxiety-related behavior and brain gene expression in the short-term (Caulfield et al., 2017). This disparity suggests that allergen exposure during development, which causes immediate and significant AI, mucus and collagen buildup, does not have immediate effects on behavior and brain function, but rather, that long-term allergic asthma symptoms that persist during development and adulthood may eventually affect later behavior and brain function.

While developmental allergen exposure caused several changes in the above behavior and brain gene expression profiles, the experimentally-induced, repeat, acute bronchoconstriction events during development had fewer long-term effects on behavior and brain gene expression. Exposure to inhaled MCH once per week, which caused significant increases in LB and Penh values at the time of exposure, only led to one long-lasting effect on behavior and no long-lasting effects on gene expression in the current study. The long-lasting behavioral effect of peri-adolescent LB was increased anxiety-related behavior on the EPM for mice that were low USV-callers (i.e., low fear) as neonates. In a prior study, we found that developmental LB led to significant short-term changes in anxiety-related behavior and brain gene expression; specifically, developmental MCH exposure caused decreased open arm time on the EPM, decreased brainstem *SERT* expression, and increased hippocampal *5Htr1a* and *Crhr1* expression 3 weeks after final MCH exposure (Caulfield et al., 2017). This difference in results between the current and prior study suggest that repeat exposure to acute LB events during development may lead to significant short-term changes in anxiety-like behavior and brain gene expression, and that these effects subside over time. Long-term anxiety-like behavior may only persist in individuals that initially show relatively low levels of fear. Overall, the results of the current study suggest that the strongest long-term impacts of developmental asthma on behavior and brain function may depend on persistent effects to lung function that result from chronic allergen exposure during development, as opposed to long-term behavior/brain changes that result from a discrete developmental period of allergic asthma symptoms.

In the current study, we also documented significant effects of sex and neonatal USV rates on adult lung and anxiety-related outcomes. Females showed more signs of lung inflammation and *IL-4* and *IL-5* expression than males—an effect that has been previously documented (Blacquière et al., 2010; Caulfield et al., 2017). Females also displayed greater exploration (i.e., faster latency to approach a novel object) compared to males. Females also had more *5HTr1a* expression in the PFC compared to males. It has been previously noted that females have higher rates of anxiety- and mood-related disorders compared to males (Roza et al., 2003; Ross et al., 2007; Holder and Blaustein, 2014). Some of these female-specific results of the present study were also evident in adult mice that, as pups, had displayed less fear-related USVs when isolated. Adult mice that displayed low-calling USV rates as pups had increased *IL-5* expression in the lungs. Pup ultrasonic calling rates also modulated some of the effects of developmental allergen and LB on adult behavior. Regardless of sex, mice that experienced weekly MCH exposures spent more time and made more entries onto the open arms of the EPM if they were high callers rather than low callers. Additionally, low-calling mice approached a novel object faster than high-calling mice in the Novel Physical test. USVs are vocal signals produced by pups in various ethologically important circumstances including isolation or separation from the nest, and they are important in mother-pup communication in early life (Bell et al., 1972; Branchi et al., 2001). In rodents, USVs can be predictors of later-life emotion regulation; mice selectively bred based on pup frequency of USVs develop into distinct high and low-calling lines, and offspring of low-calling lines demonstrate less anxiety-like behavior compared to the high-calling line (Dichter et al., 1996; Brunelli et al., 1997). In 7-day old mice, USV production can be modulated by anxiolytic and anxiogenic drugs (Fish et al., 2004; Takahashi et al., 2009). The present results are in contrast to what would be expected—if high rates of USVs are suggestive of a higher predisposition to become anxious, it would be expected that those mice would demonstrate higher levels of inflammation and anxiety-related behavior compared to low-calling mice. However, in the current study, mice that showed low-calling rates demonstrated higher levels of lung *IL-5* gene expression, more anxiety-related behavior, longer latencies to approach novelty and increased hippocampal *GR* gene expression. These results are in the opposite direction of what is expected, and they suggest that low-callers, when faced with a challenge, increase their inflammatory and anxiety-like symptoms. On the other hand, high-calling mice may have more resources to respond to developmental challenges and show fewer long-term adult consequences of adolescent stressors.

### Persistent Alteration to Lung Function After Developmental Immune Challenges

Previous research has demonstrated how early life respiratory events alter later life lung function. For example, in mice, neonatal exposure to high concentrations of oxygen causes changes in lung development that persists into adulthood (Yee et al., 2009). Human data also indicate that children who have persistent or severe asthma are more likely to continue experiencing irregular lung function as adults and are at higher risk for developing COPD (Pasterkamp et al., 1997; Tai et al., 2014b). Additionally, children that experience pneumonia in early life (before 3 years of age) have impaired lung function in adolescence and adulthood compared to subjects that never had pneumonia during this time (Chan et al., 2015). In the current study, persistent lung alterations following peri-adolescent allergen exposure may have accounted for increased immobility in the FST, a classic test for depression-related behaviors (McCormick and Green, 2013). HDM-exposed mice still showed significantly elevated lung inflammation, mucus and collagen levels at the time of FST testing, which occurred 3 months after termination of allergen exposure. More rapid and frequent immobility in the FST was likely an effect of the persistent AI and associated decreased oxygen availability for HDM-exposed mice, as opposed to “depression-like” symptoms *per se*.

Other studies have demonstrated lasting airway inflammatory processes in rodent asthma models. For example, LACK peptide (a novel antigen) exposure used to induce asthma symptoms in BALB/cAnN mice, beginning at 6 weeks of age, led to inflammatory symptoms that persisted 5 and 8 weeks after the termination of antigen exposure (Julia et al., 2002). In adult female BALB/cJ mice, intranasal exposure to ovalbumin for 12 weeks led to significant eosinophilic inflammation, goblet cell hyperplasia and collagen deposition that resolved 4 weeks after final allergen exposure, and lymphocyte inflammation and smooth muscle thickening took 8 weeks to resolve (Alrifai et al., 2014). Adult female BALB/cJ mice exposed to ovalbumin periodically over a 55-day protocol demonstrated significant inflammation but no airway hyper-responsiveness 1 month after ovalbumin exposure was terminated (McMillan and Lloyd, 2004). Adult female BALB/c mice exposed to ovalbumin every other day, over an 8-week period, had lasting inflammation 2, 4, 6 and 8 weeks following the termination of allergen exposure (Temelkovski et al., 1998). In the present study, we demonstrated that 8 weeks of peri-adolescent exposure to HDM led to persistent AI 11.5 weeks after the end of allergen exposure, and that this inflammation was more pronounced in females compared to males. These results were evident in histological measures and in cytokine-related gene expression. These persistent effects are notably longer than previously documented persistent effects in adult mice, suggesting that allergic processes that develop during childhood/adolescence may take longer to resolve than allergic processes that begin in adulthood.

In the present study, we demonstrate that lung inflammation, mucus, collagen and allergic cytokine gene expression (*IL-4* and *IL-5*) are increased in adult mice 3 months after the completion of chronic developmental allergen exposures. We have previously documented more enhanced increases in inflammation, mucus and lung gene expression 3 weeks after the completion of asthma symptom exposure, but in this prior study there was no collagen buildup at this early time point (Caulfield et al., 2017). In the current study, airway remodeling, as indicated with collagen buildup, was evident 3 months after completion of asthma symptom exposures. These results suggest that some aspects of lung function (inflammation, mucus, gene expression) persist for a long time after allergen exposure, while aspects related to lung structure (collagen build-up) require a longer time to fully form (Tanaka et al., 2002; Antunes et al., 2010; Salmond et al., 2012).

### Limitations and Future Directions

One limitation of the current study involves the dosing of MCH between the LB and the AI+LB treatment groups. In order to create similar LB and Penh values and to be conscious of humane endpoints for the mice in the MCH administration sessions, the dose of MCH (the bronchoconstriction agent) was halved in mice exposed to both HDM and MCH (i.e., the AI+LB group). Based on results from the current study, it appears that in the AI+LB group, lung inflammation, gene expression and behavior were qualitatively different from groups that received only AI or only LB. Although, we found very few statistical interactions of AI and LB, it is important to note that the difference in MCH dosing in the AI+LB group may limit the interpretation of results. Future work should establish a better treatment protocol to induce both AI and LB to understand synergistic effects of these asthma symptoms.

Another potential limitation in the present study involved the 2-min isolation method used to measure neonatal USV rates. While brief isolation causes a stress response in the pup, all pups experienced the same procedure, which was conducted prior to manipulation of asthma symptoms. It is possible that this early-life stress experience, prior to asthma manipulations, may have masked or accentuated the asthma effects reported here. However, this procedure allowed us to control for pre-asthma anxiety-related behavior in individual mice and to determine if early asthma symptoms lead to elevated anxiety-like symptoms in individuals that are otherwise predisposed toward anxiety. Further, we also used multiple behavioral outcome measures in the current study. For an initial exploratory study on potential long-term behavioral effects of allergic asthma, we felt it was important to include multiple behavioral outcomes. However, it is important acknowledge that mouse responses to the latter tests (e.g., forced swim and novelty exploration) may have been affected by earlier testing experiences.

The findings from this study have important implications for research on asthma therapy as it relates to anxiety- and depression-related disorders. This is particularly true for children and adolescents that mature with asthma symptoms and inflammation and need to treat symptoms with chronic pharmaceutical regimes. Specific asthma treatments may differentially influence mechanisms associated with anxiety and/or depression, and these effects should be evaluated in pre-clinical research. For example, many asthma patients are treated with daily inhaled corticosteroids to control asthma symptoms, and these treatments are effective in reducing inflammation (Lee et al., 2008; Alrifai et al., 2014). These drugs also lead to lasting effects on growth, adrenal function, and other processes (Merkus et al., 1993; Hanania et al., 1995; Molimard et al., 2008). However, little is known about how these treatments may affect internalizing disorder susceptibility and associated mechanisms. The current study provides a model in which to test the long-term influence of asthma treatments on both peripheral lung processes and more centralized mechanisms associated with anxiety- and depression-related symptoms. With this initial study completed, future studies can include fewer extraneous control measures in order to minimize potential confounds of early life stress effects on the developing immune system. With fewer early stressors, future studies can avoid experimental noise and provide a stronger signal for interpretation.

## Conclusion

In summary, the current study is the first to show that persistent lung inflammation coincides with changes in brain gene expression that are associated with emotion and stress regulation, providing potential mechanisms by which developmental asthma may increase risk for internalizing disorders in a rodent model. This study also demonstrates that adolescent allergen-induced lung inflammation, mucus and collagen buildup persist several months after termination of allergen exposure. An important caveat is that these long-term lung, brain and behavior responses to developmental allergic asthma may differ for males and females and may also differ depending on early life temperament/traits. Future work is required to further identify and test potential mechanisms, to determine the influence of asthma treatments, and to identify processes that predispose some individuals with developmental asthma to internalizing disorders.

## Author Contributions

JC and SC performed statistical analyses and drafted the manuscript. MC, AA, RBourne, NC, TC and LK made editorial contributions to the manuscript. SC, NC, RBourne and MC were involved in data collection. AA, TC, LK, RBonneau and SC contributed ideas to the research funding proposal and research design.

## Conflict of Interest Statement

The authors declare that the research was conducted in the absence of any commercial or financial relationships that could be construed as a potential conflict of interest.

## Abbreviations

AI: Airway inflammation
AI+LB: Airway inflammation+labored breathing
CON: Control
HDM: House dust mite
IL: Interleukin
LB: Labored breathing
MCH: Methacholine
P: Postnatal day
USV: Ultrasonic vocalization.
